# Residential Mobility Among Elementary School Students in Los Angeles County and Early School Experiences: Opportunities for Early Intervention to Prevent Absenteeism and Academic Failure

**DOI:** 10.3389/fpsyg.2019.02176

**Published:** 2019-10-10

**Authors:** Gabrielle Green, Amelia DeFosset, Tony Kuo

**Affiliations:** ^1^Division of Chronic Disease and Injury Prevention, Los Angeles County Department of Public Health, Los Angeles, CA, United States; ^2^Department of Family Medicine, David Geffen School of Medicine at UCLA, Los Angeles, CA, United States; ^3^Department of Epidemiology, UCLA Fielding School of Public Health, Los Angeles, CA, United States; ^4^Population Health Program, UCLA Clinical and Translational Science Institute, Los Angeles, CA, United States

**Keywords:** residential mobility, school connectedness, chronic absenteeism, elementary school, academic success

## Abstract

School connectedness is closely linked to academic success: students who are engaged at school have better attendance and academic performance, and are less likely to drop out. Residential mobility – having moved homes – can increase the risk of a negative academic trajectory (e.g., absenteeism and academic failure). Increasing housing instability in the United States due to rising housing costs, especially in urban areas, has made residential mobility a growing concern. While existing research has examined residential mobility among students and its connection to long-term consequences such as absenteeism and academic failure, less is known about how residential mobility relates to potential intermediate school experiences (e.g., school disconnectedness, low perceived academic ability, and experiences with school violence and harassment) that contribute to a negative academic trajectory. This study examines associations between residential mobility in elementary school and school experiences in a large urban jurisdiction. Data were collected from a sample of public elementary school students in Los Angeles County (5th grade, *n* = 5,620) via the California Healthy Kids Survey (2013–2014). Descriptive, Chi-square, multiple logistic regression analyses, and predicted probabilities were performed to examine the relationships between past-year residential mobility and indicators of school connectedness and school-based relationships, perceived academic performance, and exposure to violence and harassment. More than a third (36.6%) of students in the analysis sample moved at least once in the past year. After adjusting for neighborhood and family factors, a higher number of past-year moves was significantly associated with poorer school experiences, including lower odds of school connectedness for high-movers (2+ moves) [adjusted odds ratio (AOR) = 0.77; 95% confidence interval (CI) = 0.68–0.86], compared to non-movers. Movers had lower odds of perceived academic ability (1 move: AOR = 0.72; CI = 0.63–0.83; 2+ moves: AOR = 0.55; CI = 0.44–0.69), but higher odds of exposure to violence and harassment as a victim (1 move: AOR = 1.26, CI = 1.17–1.37; 2+ moves: AOR = 1.34, CI = 1.17–1.54), and as a perpetrator (1 move: AOR = 1.21, CI = 1.08–1.36; 2+ moves: AOR = 1.54, CI = 1.24–1.92). These results highlight the value of developing and implementing strategies that can identify and support students who move at young ages, to prevent student disengagement and promote attendance and academic success early in their life trajectory.

## Introduction

Regular school attendance, and the closely and reciprocally linked issue of school connectedness, strongly predict academic success (Centers for Disease Control and Prevention, 2018). However, an estimated 7 million youth are chronically absent each year (missing 15 or more school days), representing 14–20% of elementary and secondary school students, respectively (Department of Education, 2019). School disconnectedness is the perception that adults and peers at school do not care about a student’s academic and personal well-being (Ashley et al., 2012). School disconnectedness often manifests alongside absenteeism (Blum, 2005), which in turn correlates with academic failure and dropout (National Collaborative on Education and Health, 2015), and a number of interrelated risk behaviors including involvement in bullying, frequent discipline problems, and substance use (Gastic, 2008). Existing conceptual models suggest that school attendance, connectedness, and academic success result from the interaction of factors related to the child, peers, school, family, and community (Freudenberg and Ruglis, 2007; Kearney, 2008; Tyler and Lofstrom, 2009; Gee and Krausen, 2015). Since educators have limited power to intervene on community and family factors that undermine academic success, anticipating and promptly recognizing their impacts represent a critical approach to prevent or mitigate harm. This paper will focus on one such factor: residential mobility.

Residential mobility – having moved homes – can be detrimental to academic success, even when the student remains at the same school (Voight et al., 2012). Residential mobility is inversely associated with school readiness (Ziol-Guest and McKenna, 2014), attendance (Ersing et al., 2009), academic performance (e.g., test scores, grade point averages), grade progression, and graduation (Scanlon and Devine, 2001). Approximately 11% of youth aged 1–17 move homes in a given year (U.S. Census Bureau, 2018). Whether a family moves for a positive reason (e.g., a new job, larger home, or safer neighborhood) or a negative one (e.g., divorce, job loss, or housing instability), moving can be a disruptive and stressful event in a child’s life (Coulton et al., 2012; Mollborn et al., 2018). Residential mobility is higher among low-income individuals, renters, and racial/ethnic minority groups (Jelleyman and Spencer, 2008), suggesting socioeconomic vulnerability plays a role. With housing costs on the rise, particularly for renters (Sparshott, 2015), it is possible that residential mobility may also increase in coming years. Identifying the early warning signs exhibited by “movers” could allow schools to provide targeted supports before attendance falters and students start to struggle academically. The pathways through which residential mobility may ultimately influence academic failure, and the contribution of likely intermediate factors such as school disconnectedness, have not been fully described in the literature, prompting calls for additional work in this area (Scanlon and Devine, 2001; Jelleyman and Spencer, 2008; Anderson et al., 2014).

This study examined the relationships between residential mobility and potential cognitive and behavioral precursors to absenteeism and academic failure that have been less explored in the literature. A primary goal was to provide preliminary information that could aid researchers in conceptualizing and testing more nuanced pathways for how residential mobility impacts youth academic success, while also generating findings that could guide prevention strategies among youth-serving institutions, especially schools. To that end, this study uses data from a large, sample of elementary school students to analyze the associations between level of residential mobility and school experiences such as school connectedness, perceived academic ability, and exposure to violence and harassment. Although a body of literature speaks to the strong relationship between these school experiences and poor academic outcomes, including absenteeism and academic failure, research has not thoroughly examined their potential role in the pathways linking residential mobility to academic outcomes (Gasper et al., 2010; Voight et al., 2017). Furthermore, the way these processes play out at younger ages has been relatively less characterized in the literature (Lawrence et al., 2015; Beck et al., 2016), despite findings that younger students are more vulnerable to the negative effects of moving (Scanlon and Devine, 2001), and the likelihood that problems encountered in elementary school will compound over time (Lawrence et al., 2015).

## Materials and Methods

### Theoretical Framework

**FIGURE 1:**
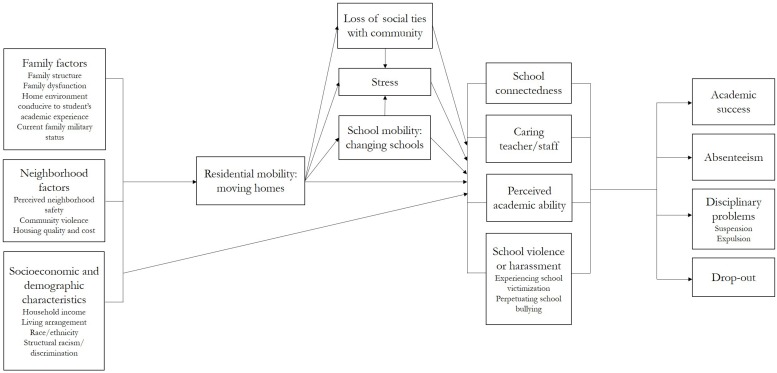
Theoretical framework of the relationships between residential mobility and school experiences among elementary school students.

The study team developed a theoretical framework to guide the present analysis, depicting the relationship between residential mobility among elementary school students and their experiences at school (Figure 1). Building upon prior research, this framework centers on the hypothesis that students who have moved are more likely to have poorer school experiences, and this association may be heightened among students who have more exposure, compared to students who have less exposure, to residential mobility.

This framework posits that a broad set of neighborhood, family, and socioeconomic/demographic factors influence whether youth experience residential mobility. Neighborhood conditions, such as perceived safety, level of neighborhood crime or violence, and housing quality and cost, can be driving forces behind a residential move. Family dynamics can also provoke a residential move, due to changes to family structure (e.g., a divorce) or family dysfunction (e.g., physical or emotional abuse, substance use) (Astone and McLanahan, 1994; Dong et al., 2005). A family’s current military status is also linked to residential mobility: youth in military families are more likely to change residences than youth in civilian families (Child and Family Research Partnership, 2017). Regarding socioeconomic and demographic factors, low-income families may be more vulnerable to fluctuations in housing costs, and therefore be more likely to move, often to a substandard residence (e.g., housing that is unsafe and unhealthy to live in) (Skobba and Goetz, 2013; Kang, 2019) or an unstable living arrangement (e.g., a relative’s home or a shelter) (Skobba and Goetz, 2013). However, a change in household income, such as a change to a higher-paying job, could precipitate a move to a higher quality residence. Furthermore, structural factors such as racism and discrimination may contribute to the high rates of residential mobility seen among non-white youth (Turner and Ross, 2005; Acevedo-Garcia et al., 2008; Perkins, 2017). It is also well documented that the factors identified in this theoretical framework as influencing the likelihood of a residential move – neighborhood conditions, family dynamics, a household member currently serving in the military, and socioeconomic/demographic characteristics (e.g., household income, structural racism/discrimination) – independently influence a student’s school experiences (Woolley and Grogan-Kaylor, 2006; Spriggs et al., 2007; Felix et al., 2009; Mmari et al., 2010; Herbers et al., 2012; Anderson et al., 2014; U.S. Department of Housing and Urban Development, 2014; Low et al., 2017; National Education Association, 2019).

When these factors lead to residential mobility, the act of moving homes can immediately impact youth. The move itself can be a stressful event (e.g., parents may be under strain during the transition from the old home to the new, and/or youth may be emotional about leaving their old home) (Murphey et al., 2012; Mollborn et al., 2018). In addition, the move from a familiar neighborhood can lead to a loss of social ties with that community (Anderson et al., 2014). Finally, the change in homes may also cause school mobility – a change in schools. These direct outcomes of residential mobility may subsequently influence youths’ school experiences in three key ways. First, increased stress, loss of previous community-based relationships, and/or changing schools could all erode school-based relationships. Youth could pull away from peers and adults at school or struggle to reestablish these relationships at a new school, undermining both a broader sense of school connectedness and relationships with caring teachers or staff. Second, students who move may also experience a disruption to their study habits or routines, potentially causing academic performance to falter (even briefly), which could lead to lower perceived academic ability, or confidence in their school work. Third, students who have moved may be more likely to be exposed to violence or harassment at school – either as a victim or as a perpetrator. If students who move are less connected to individuals at school, feel less frequently supported by teachers, and struggle more academically, it follows that these students may also be more vulnerable to being the victim of violence or harassment from other students, and may also be more likely to act out and instigate bullying (Gasper et al., 2010).

A robust literature base supports the associations between the school experiences examined in this study (school connectedness, relationships with caring teachers and staff, perceived academic ability, and experiences with school-based violence and harassment) and academic outcomes for youth: students who have poor school experiences may be at a higher risk of absenteeism, poor grades, suspension/expulsion, or drop-out (Voight et al., 2012). Students who feel connected to school are more likely to attend school regularly, earn good grades, avoid disciplinary problems such as school suspension, and to stay in school through graduation (Centers for Disease Control and Prevention, 2009; Sheryl et al., 2014). Positive teacher–student relationships have been linked to these same outcomes, as well as a lower incidence of behavioral problems, as students feel more supported at school and more motivated to learn (Quin, 2017). Students with confidence in their academic ability are more likely to earn good grades (Marsh and Martin, 2011). Finally, school-based violence and harassment are negatively linked to students’ academic outcomes: students who are bullied are more likely to have increased absenteeism (Steiner and Rasberry, 2015) and poor grades (Juvonen et al., 2011; Ladd et al., 2017), while students who bully others are more likely to drop out of school (U.S. Department of Health and Human Services, 2019).

### Instrument

The current study uses student-level data from the 2013 to 2014 elementary school version of the California Healthy Kids Survey (CHKS). Developed by WestEd in conjunction with the California Department of Education (CDE), the CHKS is designed to provide information regarding risk behaviors and protective factors among California’s school-age population. Questions are largely adapted from the Biennial California Student Survey and the Youth Risk Behavior Survey, which measure similar constructs at a national level (WestEd, 2019). Available annually to school districts in California, the CHKS comprises an elementary school version targeted to fifth grade students, a middle school version for seventh grade students, and a high school version that can be administered to ninth and eleventh grade students. The elementary CHKS includes a mandatory core module, as well as six optional supplemental modules centered on targeted topics; districts may also elect to design a custom module (California Healthy Kids Survey, 2019). It is voluntary for districts to administer the CHKS and there is a fee to do so. However, there are some cases in which districts receive funding that specifically require (and financially support) CHKS administration, such as the Title IV Safe and Drug-Free Schools and Communities program, the Safe and Supportive Schools grant, and the Tobacco Use Prevention Education program (Adams, 2013; Austin, 2013; California Department of Education, 2019).

California Healthy Kids Survey data have been used in numerous research studies to examine topics such as: substance use and/or exposure to violence and harassment (Wong et al., 2004; Felix et al., 2009; Russell et al., 2012; Gilreath et al., 2014b; Bostean et al., 2015), military-connected youth risk behaviors (Gilreath et al., 2013, 2014a; Cederbaum et al., 2014; Sullivan et al., 2015), school health center use (Amaral et al., 2011; Stone et al., 2013; Lewis et al., 2015), gang membership (Estrada et al., 2013; Lenzi et al., 2015), asthma prevalence (Davis et al., 2006, 2007), school climate (O’Malley et al., 2015), television and video game habits (Armstrong et al., 2010), and gender identity (Perez-Brumer et al., 2017), primarily using cross-sectional observational study designs. The study team collaborated with WestEd in 2015 to obtain data for all students in the county who completed the CHKS between 2000 and 2015, to inform planning of school-related health and wellness initiatives.

### Administration and Sampling

The CHKS is designed to be administered either in print or online at the school site (additional details on sampling are provided below), typically during the fall or spring. For the elementary CHKS, active parental consent is required; a student who does not turn in a written permission form from a parent or guardian will not be administered the CHKS. The survey does not collect identifying information, and students and their families are informed that responses are anonymous.

WestEd provides districts with guidelines on how to survey at the school and student level to generate results that are maximally representative of the target grade level. Participating districts are advised to survey *all* students in a selected grade level if either of the following criteria is met: (a) the district has 10 or fewer schools with that selected grade level, or (b) the district has 900 or fewer enrolled regular students at the selected grade level. If neither criterion is met, the district is eligible to randomly sample students in consultation with a technical advisor from WestEd, however, sampling is not required (Austin et al., 2013). In addition, the survey should be administered during an appropriate class period (determined in consultation with WestEd), such as a required class attended by all enrolled students in the selected grade, and 100% of selected classrooms should participate. Following data collection, WestEd provides data quality standards to gauge the representativeness and validity of collected data. In addition to following the appropriate survey strategy (based on sampling criteria described above), data were considered sufficiently valid in 2013–2014 (the study year, see below) if: (a) 70% or more of parents in the selected sample completed the consent form, or (b) 60% or more of students in a participating grade returned a complete and usable questionnaire. A 70% response rate was considered good, and 60–69% was considered acceptable, but borderline (Austin et al., 2013).

The present study represents a secondary analysis of Los Angeles County’s elementary school CHKS dataset (as described above), focusing on the 2013–2014 academic year, which is the most recent year that a question on residential mobility was available. The study team conducted a two-stage review and selection process to develop the analysis sample. First, the study team conducted a confirmatory review to ensure all data met basic parameters for participating in the elementary CHKS: (1) it was collected from a public school district with elementary grades, (2) from students in the fifth grade (encompassing ages 9–12), and (3) in Los Angeles County. Additionally, to maximize comparability across educational contexts, charter schools were excluded during this stage. During the second stage, district-level data were reviewed to assess adherence to minimum quality standards, as outlined by WestEd (see above). Because district-level sampling plans were not available to the study team, districts were first categorized as non-sampling eligible or sampling eligible (based on publicly available data from CDE regarding number of schools and student enrollment during the study year). Non-sampling eligible districts (where 100% of students should have been surveyed) were excluded if they did not achieve a response rate of at least 60% of enrolled students (the minimum threshold for data to be classified as acceptable by WestEd). For sampling eligible districts, it was assumed that an approved sampling plan was followed.

### Measures

The following measures from the elementary CHKS were selected due to their alignment with the study’s theoretical framework.

#### Residential Mobility

Residential mobility was assessed using the single question, “During the past year, how many times have you moved (changed where you live)?” Response options were: “0 times,” “1 time,” and “2 or more times.” In this study, students were categorized as non-movers (those that answered “0 times”), low-movers (“1 time”), or high-movers (“2 or more times”).

#### Family and Neighborhood Factors

Perceived neighborhood safety was examined through one question: “Do you feel safe outside of school?” Response options were: “never,” “some of the time,” “most of the time,” and “all of the time.” A dichotomous variable was created (never/some versus most/all). A home environment conducive to the student’s academic experience was examined through one question: “Does a parent or some other grown-up at home care about your schoolwork?” Response options were: “never,” “some of the time,” “most of the time,” and “all of the time.” These responses were collapsed into a dichotomous variable (all versus most/some/never). Current family military status was measured through one question: “Is your father, mother, or caretaker currently in the military?” Response options were “no,” “yes,” and “don’t know.” Responses of “don’t know” were coded as missing.

#### School Experiences

##### School connectedness

School connectedness was measured using a scale developed by WestEd that was adapted from the National Longitudinal Study on Adolescent Health (Austin et al., 2013; WestEd, 2014). The scale was constructed using responses to five questions: “Do you feel close to people at school?,” “Are you happy to be at this school?,” “Do you feel like you are part of this school?,” “Do teachers treat students fairly at school?,” and “Do you feel safe at school?” Response options for all questions were “never,” “some of the time,” “most of the time,” and “all of the time”; which were numerically coded as 1, 2, 3, and 4, respectively, and summed to obtain the scale value for school connectedness, with higher values representing greater school connectedness. Cronbach’s alpha for the scale was 0.69. The scale value was divided by five to obtain an average question response score. A dichotomous variable was developed to measure a high level of school connectedness compared to a moderate/low level, based on parameters used by WestEd: respondents were labeled as “high” if their average question response score was greater than three, while students with an average less than or equal to three were labeled as “moderate/low.”

##### Caring teacher/staff

The presence of caring teachers or school staff was examined through one survey item: “At my school, there is a teacher or some other adult who really cares about me.” Response options were: “never,” “some of the time,” “most of the time,” and “all of the time.” Responses were dichotomized as never/some of the time and most/all of the time.

##### Perceived academic ability

Perceived academic ability was measured using a question designed to assess achievement among elementary-aged students: “How well do you do in your schoolwork?” Answer choices were: “I’m one of the best students,” “I do better than most students,” “I do about the same as others,” and “I don’t do as well as most others.” A dichotomous variable was created, in which “above average” comprised the options “I’m one of the best students” and “I do better than most students,” while “average or below” represented “I do about the same as others” and “I don’t do as well as most others.”

##### School violence or harassment

Three types of exposure to violence and harassment as a victim in the past year were assessed separately. First, respondents were asked, “Do other kids hit or push you at school when they are not just playing around?” and “Do other kids at school spread mean rumors or lies about you?” For both questions, the answer choices were: “never,” “some of the time,” “most of the time,” and “all of the time.” Responses were dichotomized as “yes” (comprising “some of the time,” “most of the time,” and “all of the time”) and “no” (“never”). The third question was: “Have other kids at school ever teased you about what your body looks like?” Answer choices were “yes” or “no.”

Two types of exposure to violence and harassment as a perpetrator in the past year were examined through two separate questions. Students were asked, “During the past year, how many times have you hit or pushed other kids at school when you were not playing around?” and “During the past year, how many times have you spread mean rumors or lies about other kids at school?” For both questions, the answer choices were: “0 times,” “1 time,” “2 times,” or “3 or more times.” Responses to each question were converted into a dichotomous variable: “yes” (comprising “1 time,” “2 times,” and “3 or more times”) and “no” (“0 times”).

### Data Analysis

First, descriptive statistics were generated to characterize the distribution of variables of interest in the sample. Second, Chi-square analyses examined bivariate associations between residential mobility and all other analysis variables: school connectedness, caring teachers/staff, perceived academic ability, exposure to violence and harassment, perceived neighborhood safety, presence at home of an adult who cared about the student’s schoolwork, and current family military status. Third, multiple logistic regression analyses examined the relationships between residential mobility and school experiences, controlling for perceived neighborhood safety, presence at home of an adult who cared about the student’s schoolwork, and current military status. The regression models were adjusted for clustering to account for the potential correlation of responses by school district. Fourth, to facilitate interpretation of regression results, post-estimation analyses were conducted. Specifically, the predicted probability for each logistic regression was calculated using the sample means of the control variables; this approach adjusts for any systematic differences in these covariates. All analyses were performed using Stata 15.1 (Stata Corp. LP, College Station, TX, United States). The study was deemed exempt from review by the Institutional Review Board of the Los Angeles County Department of Public Health.

## Results

### Sample

#### Sample Districts

**FIGURE 2:**
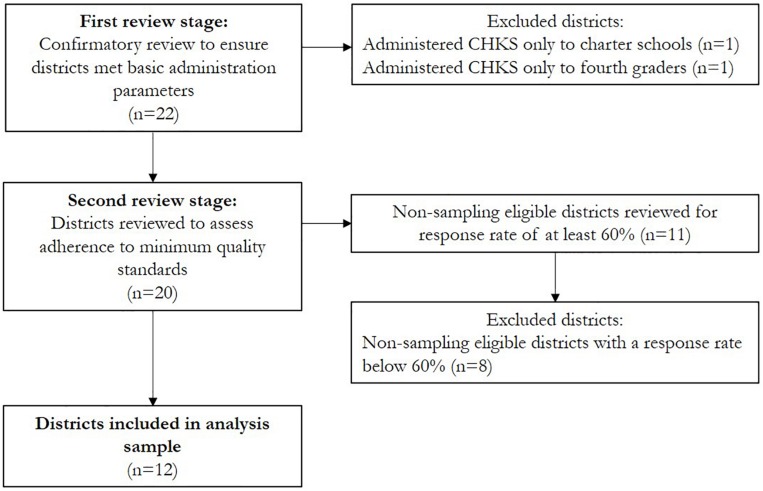
Flowchart of the inclusion process for districts in the analysis sample.

Twenty-two public school districts in Los Angeles County participated in the elementary CHKS in 2013–2014, out of 67 eligible districts (i.e., districts that had at least one elementary school). Figure 2 summarizes how districts were selected into the analysis sample through the two-stage review and selection process. Two districts were excluded during the first review stage: one district only administered the CHKS to charter schools, while the other district only administered the survey to fourth graders. The second review stage focused on adherence to minimum data quality standards; nine districts were categorized as sampling eligible, and were therefore included in the analysis sample. The remaining 11 districts were categorized as non-sampling eligible, thus requiring a response rate of 60% or above; 8 districts fell below this threshold and were excluded. The final analysis sample therefore had 12 districts.

To provide additional context, district-level characteristics that are known to be related to residential mobility and/or risk behaviors impacting academic success (Voight et al., 2012, 2017; Metzger et al., 2015), but that were not captured at the student level by the CHKS, were examined in comparison to all other districts in Los Angeles County that had at least one school with a fifth grade class (Table 1). The 12 districts in the analysis sample were somewhat smaller (lower mean total enrollment and a lower mean fifth grade enrollment) than the 64 other districts, likely because the most populous school district in the county was not part of the sample (with a total district enrollment eight times bigger than the next most populous district). In terms of fifth grade demographics, analysis sample districts had a slightly higher proportion of non-Hispanic white students, and a lower proportion of Hispanic/Latino and Asian students. Enrollment in the free/reduced price meal program was about 10 percentage points lower in the analysis sample districts. The truancy rate was slightly lower in the analysis sample districts. For districts with high school grades (grades 9–12), the adjusted high school drop-out rate was marginally higher in analysis sample districts.

**TABLE 1:** Characteristics of school districts participating in the 2013–2014 elementary California Healthy Kids Survey and school districts not in the analysis sample, Los Angeles County^1,2,3,4^.

	**CHKS school districts in analysis sample (*n* = 12)**			**School districts not in analysis sample (*n* = 64)**		
**Characteristic**	**Mean (*SD*)**	**Min**	**Max**	**Mean (*SD*)**	**Min**	**Max**
**Total enrollment**						
Entire district	14,172(8,574)	3,132	29,951	20,570 (81,191)	253	653,826
5th grade only	1,030(632)	241	2,136	1,595 (6,186)	35	49,885
**Percentage of 5th grade students in race/ethnic category**						
Hispanic/Latino	50.2 (40.2)	6.8	98.1	55.5 (26.2)	9.7	97.7
Non-Hispanic White	28.6 (29.3)	0.2	74.8	22.4 (22.2)	0.3	80.6
Asian	7.4 (8.3)	0.0	27.4	11.6 (17.6)	0.0	66.3
Black/African-American	4.2 (5.7)	0.2	18.6	5.6 (7.7)	0.0	40.0
Two or more races	3.7 (4.9)	0.0	15.2	2.2 (2.5)	0.0	11.4
American Indian or Alaskan native	4.4 (14.8)	0.0	51.5	0.2 (0.3)	0.0	1.1
Filipino	1.6 (2.0)	0.0	6.4	2.1 (2.1)	0.0	11.4
Pacific Islander	0.4 (0.4)	0.0	1.3	0.3 (0.3)	0.0	1.9
Not reported	0.08 (0.2)	0.0	0.5	0.7 (0.9)	0.0	4.0
**Percentage of 5th grade students qualifying for free/reduced price meals**	49.9 (39.8)	2.9	93.6	59.4 (26.5)	1.4	99.7
**Truancy rate^5^**	18.4 (11.2)	6.0	31.7	22.9 (14.1)	0.0	57.3
**Adjusted drop-out rate, grades 9–12^6^**	3.2 (4.7)	0.0	14.8	2.5 (3.5)	0.0	16.0

*^1^Data publicly available from the California Department of Education website (https://www.cde.ca.gov) for the year 2013–2014. ^2^“School districts not in analysis sample”: Los Angeles County public school districts with 1 + fifth grade class in 2013–2014, but either did not administer the elementary CHKS that year or did not meet this study’s inclusion criteria. ^3^Values for count variables are rounded to the nearest whole number. Percentages may not add to 100 due to rounding. ^4^Some variables include fewer districts, either due to unavailable data or non-applicability of the variable (i.e., adjusted drop-out rate is for grades 9–12; as such, districts that do not have these grades are not included). ^5^Data represent the district-wide truancy rate, which may include schools that do not serve 5th graders. Details on how the truancy rate is calculated are available on the California Department of Education website. ^6^The adjusted drop-out rate is calculated for districts that have high school grades (grades 9–12). Details on how the adjusted drop-out rate is calculated are available on the California Department of Education website.*

#### Sample Students

The characteristics of the analysis sample are presented in Table 2. In total, 7,230 fifth grade students met criteria for inclusion in the analysis sample. A further 1,610 respondents were excluded from the analysis because they had missing data for any of the variables of interest (using a listwise deletion approach), resulting in a final analysis sample containing 5,620 student respondents across the 12 school districts.

**TABLE 2:** Chi-square associations between residential mobility status and school experiences, family and neighborhood experiences, and student demographics, among respondents of the elementary California Healthy Kids Survey in Los Angeles County public school districts, 2013–2014^1^.

	**Full sample (*n* = 5,620)**	**Non-mover (*n* = 3,566)**	**Low-mover (*n* = 1,218)**	**High-mover (*n* = 836)**	
	**Column %**	**Column %**	**Column %**	**Column %**	***p*-value**
**School experience**					
**School connectedness**					
High level	68.8	71.0	66.9	62.3	< 0.001^*⁣**^
Moderate/low level	31.2	29.0	33.1	37.7	
**Teacher or staff cares about student**					
All or most of the time	85.7	86.7	84.8	82.5	0.006^∗∗^
Sometimes or never	14.3	13.3	15.2	17.5	
**Perceived academic ability**					
Above average	49.3	53.6	44.9	37.3	< 0.001^*⁣**^
Average or below	50.7	46.4	55.1	62.7	
**Exposure to violence or harassment (victim)**					
Been hit or pushed (yes)	36.7	33.9	40.3	43.4	< 0.001^*⁣**^
Had rumors or lies spread (yes)	40.2	37.8	41.4	48.4	< 0.001^*⁣**^
Been teased about body (yes)	26.3	24.6	28.0	31.3	< 0.001^*⁣**^
**Perpetrator of violence or harassment**					
Hit or pushed a classmate (yes)	28.8	26.1	30.7	37.3	< 0.001^*⁣**^
Spread rumors or lies about a classmate (yes)	22.9	20.1	26.1	30.3	< 0.001^*⁣**^
**Family and neighborhood factors**					
**Perceived neighborhood safety**					
All or most of the time	71.3	73.8	69.5	63.6	< 0.001^*⁣**^
Sometimes or never	28.7	26.2	30.5	36.4	
**Adult at home cares about student’s schoolwork**					
All the time	83.0	83.7	83.1	79.8	0.028^∗^
Less than all the time	17.1	16.4	16.9	20.2	
**Current family military status**					
Parent or caretaker in the military (yes)	9.1	7.3	10.6	14.5	< 0.001^*⁣**^

*^1^Statistically significant, ^∗^*p* < 0.05; ^∗∗^*p* < 0.01; ^∗∗∗^*p* < 0.001.*

Over a third of respondents (36.6%) reported past-year residential mobility: 21.7% of respondents were low-movers (moved once) and 14.9% were high-movers (moved two or more times), while the remaining two-thirds (63.5%) were classified as non-movers. Over two-thirds (68.8%) of respondents reported a high level of school connectedness, and most students (85.7%) felt that teachers or school staff cared about them all or most of the time. Regarding perceived academic ability, about half of students (49.3%) believed that they were performing above average. In terms of past-year exposure to violence and harassment at school as a victim, 36.7% of students reported being hit or pushed, 40.2% reported having had rumors or lies spread about them, and 26.3% said they had been teased about their body. In terms of past-year perpetuation of violence and harassment at school, 28.8% of students reported having hit or pushed a classmate, and 22.9% reported having spread rumors or lies about a classmate.

### Relationships Between Residential Mobility and School Experiences

#### Chi-Square Associations

Chi-square analyses revealed significant bivariate associations between residential mobility and all other analysis variables (Table 2). Movers had poorer school experiences than non-movers; among movers, high-movers fared worse than low-movers. Among high-movers, 62.3% had a high level of school connectedness, compared to 66.9% of low-movers and 71.0% of non-movers. High-movers also had the lowest rate (82.5%) of reporting that a teacher or staff member cared about them all or most of the time, compared to low-movers (84.8%) and non-movers (86.7%). Only 37.3% of high-movers felt that they were performing above average academically, compared to 44.9% of low-movers and 53.6% of non-movers. Both exposure to, and perpetration of, violence or harassment were higher among movers than non-movers, with high-movers having the highest rates. High-movers had rates of violence approximately 10 percentage points above non-movers: 43.4% of high-movers had been hit or pushed, compared to 40.3% of low-movers and 33.9% of non-movers. Similarly, 37.3% of high-movers reported hitting or pushing a classmate, compared to 30.7% for low-movers, and 26.1% for non-movers. Almost half (48.4%) of high-movers had rumors or lies spread about them; rates were 41.4% for low-movers and 37.8% for non-movers. Nearly a third (31.3%) of high-movers had been teased about their body, versus 28.0% for low-movers and 24.6% for non-movers. An estimated 30.3% of high-movers had spread rumors or lies about a classmate, compared to 26.1% of low-movers and 20.1% of non-movers. Family and neighborhood factors were significantly associated with residential mobility in expected directions: compared to non-movers, movers (especially high-movers) had lower rates both of feeling safe in their neighborhood and of having an adult at home that cared about their schoolwork, and had a greater rate of having a parent or caretaker currently in the military.

#### Multiple Logistic Regressions and Predicted Probabilities

Multiple logistic regression analyses examined the relationship between residential mobility and school experiences, controlling for perceived neighborhood safety, presence of an adult at home that cares about the student’s schoolwork, and current family military status (Table 3). Post-estimation tests were conducted on the above regression models to generate predicted probabilities of school experiences by mobility status at the sample means of the control variables (Table 4).

**TABLE 3:** Multiple regression model results: crude and adjusted odds ratios examining the relationships between school experiences and residential mobility among respondents of the elementary California Healthy Kids Survey in Los Angeles County public school districts, 2013–2014^1,2,3,4,5^.

	**High level of school connectedness**	**Teacher/staff cares about student all or most of the time**	**Above average perceived academic ability**	**Been hit or pushed**	**Had rumors or lies spread**	**Been teased about body**	**Hit or pushed a classmate**	**Spread rumors or lies about a classmate**
	**OR (95% CI)**	**OR (95% CI)**	**OR (95% CI)**	**OR (95% CI)**	**OR (95% CI)**	**OR (95% CI)**	**OR (95% CI)**	**OR (95% CI)**
	**AOR (95% CI)**	**AOR (95% CI)**	**AOR (95% CI)**	**AOR (95% CI)**	**AOR (95% CI)**	**AOR (95% CI)**	**AOR (95% CI)**	**AOR (95% CI)**
**Residential mobility**								
Low-mover	0.83 (0.72-0.96)^∗^	0.86 (0.73-1.01)	0.71 (0.61-0.82)^∗∗∗^	1.32 (1.22-1.42)^∗∗∗^	1.16 (1.02-1.32)^∗^	1.19 (1.08-1.31)^∗∗∗^	1.26 (1.13-1.40)^∗∗∗^	1.40 (1.05-1.87)^∗^
	0.86 (0.74-1.00)	0.90 (0.74-1.08)	0.72 (0.63-0.83)^∗∗∗^	1.26 (1.17-1.37)^∗∗∗^	1.11 (0.98-1.26)	1.14 (1.06-1.24)^∗∗^	1.21 (1.08-1.36)^∗∗^	1.36 (1.02-1.83)^∗^
High-mover	0.68 (0.59-0.77)^∗∗∗^	0.73 (0.62-0.86)^∗∗∗^	0.52 (0.41-0.64)^∗∗∗^	1.49 (1.30-1.72)^∗∗∗^	1.54 (1.32-1.80)^∗∗∗^	1.40 (1.08-1.81)^∗∗^	1.69 (1.34-2.12)^∗∗∗^	1.72 (1.38-2.15)^∗∗∗^
	0.77 (0.68-0.86)^∗∗∗^	0.82 (0.68-0.97)^∗^	0.55 (0.44-0.69)^∗∗∗^	1.34 (1.17-1.54)^∗∗∗^	1.40 (1.22-1.60)^∗∗∗^	1.26 (0.98-1.63)	1.54 (1.24-1.92)^∗∗∗^	1.59 (1.28-1.97)^∗∗∗^
**Perceived neighborhood safety**								
All or most of the time	2.32 (2.01-2.68)^∗∗∗^	1.86 (1.59-2.16)^∗∗∗^	1.60 (1.44-1.79)^∗∗∗^	0.56 (0.49-0.64)^∗∗∗^	0.54 (0.46-0.63)^∗∗∗^	0.60 (0.51-0.70)^∗∗∗^	0.75 (0.66-0.86)^∗∗∗^	0.67 (0.57-0.79)^∗∗∗^
**Adult at home cares about student’s schoolwork**								
All the time	2.61 (2.31-2.96)^∗∗∗^	2.20 (1.93-2.51)^∗∗∗^	1.41 (1.16-1.71)^∗∗^	0.68 (0.62-0.76)^∗∗∗^	0.74 (0.65-0.84)^∗∗∗^	0.77 (0.66-0.89)^∗∗^	0.59 (0.51-0.67)^∗∗∗^	0.58 (0.50-0.68)^∗∗^
**Current family military status**								
Parent or caretaker in the military (yes)	0.72 (0.59-0.88)^∗∗^	0.74 (0.60-0.90)^∗∗^	0.83 (0.62-1.09)	1.77 (1.42-2.21)^∗∗∗^	1.64 (1.45-1.85)^∗∗∗^	1.79 (1.56-2.06)^∗∗∗^	1.95 (1.56-2.44)^∗∗∗^	1.44 (1.15-1.80)^∗∗^

*^1^Statistically significant, ^∗^*p* < 0.05; ^∗∗^*p* < 0.01; ^∗∗∗^*p* < 0.001. ^2^Adjusted for clustering within school districts. ^3^Reference categories: non-mover (residential mobility), sometimes or never (perceived neighborhood safety), most of the time or sometimes or never (adult at home cares about student’s schoolwork), no (current military status). ^4^OR, odds ratio; CI, confidence interval. ^5^AOR, adjusted odds ratio.*

**TABLE 4:** Predicted probabilities of school experiences by residential mobility status among respondents of the elementary California Healthy Kids Survey in Los Angeles County public school districts, 2013–2014^1^.

	**Mobility status (%)**		
	**Non-mover**	**Low-mover**	**High-mover**
	**(%)**	**(%)**	**(%)**
**Level of school connectedness^2^**			
High	71.4	68.3	65.7
**Teacher or staff cares about student^3^**			
All or most of the time	87.2	85.9	84.7
**Perceived academic ability^4^**			
Above average	53.2	45.1	38.4
**Exposure to violence or harassment (victim)^5^**			
Been hit or pushed (yes)	34.2	39.6	41.1
Had rumors or lies spread (yes)	38.2	40.7	46.3
Been teased about body (yes)	24.6	27.2	29.2
**Perpetrator of violence or harassment^6^**			
Hit or pushed a classmate (yes)	26.1	30.0	35.3
Spread rumors or lies about a classmate (yes)	20.0	25.5	28.4

*^1^Predicted probabilities are at the sample means of the control variables: perceived neighborhood safety, presence of an adult at home that cares about the student’s schoolwork, and current family military status. ^2^In the adjusted logistic regression model, the odds of reporting a high level of school connectedness was significantly lower for high-movers than for non-movers (*p* < 0.001). ^3^In the adjusted logistic regression model, the odds of reporting that a teacher or staff member cared about them all or most of the time was significantly lower for high-movers than for non-movers (*p* < 0.05). ^4^In the adjusted logistic regression model, the odds of a student perceiving their academic ability to be above average was significantly lower for both low- and high-movers than for non-movers (*p* < 0.001). ^5^In the adjusted logistic regression model, relative to non-movers, the odds of having been hit or pushed was significantly higher for both low- and high-movers (*p* < 0.001), the odds of having had rumors or lies spread about them was significantly higher for high-movers (*p* < 0.001), and the odds of being teased about their body was significantly higher for low-movers (*p* < 0.01). ^6^In the adjusted logistic regression model, relative to non-movers, the odds of having hit or pushed a classmate was significantly higher for low-movers (*p* < 0.01) and high-movers (*p* < 0.001), and the odds of having spread rumors or lies about a classmate was significantly higher for low-movers (*p* < 0.05) and high-movers (*p* < 0.001).*

Generally, past-year moving was associated with poorer school experiences (Table 3). In adjusted regression analysis, high-movers had significantly lower odds of reporting a high level of school connectedness [adjusted odds ratio (AOR) = 0.77; 95% confidence interval (CI) = 0.68–0.86] compared to non-movers, corresponding to a 65.7% predicted probability of having a high level of school connectedness, compared to 68.3% for low-movers and 71.4% for non-movers. Similarly, high-movers also had significantly lower odds of reporting that a teacher or staff member cared about them all or most of the time (AOR = 0.82; CI = 0.68–0.97); predicted probabilities were 84.7% for high movers, 85.9% for low movers, and 87.2% for non-movers. However, neither of these relationships (school connectedness, caring teachers/staff) was statistically significant for low-movers. Compared to non-movers, both high- and low-movers had significantly lower odds of perceiving their academic ability to be above average (high-movers: AOR = 0.55; CI = 0.44–0.69; low-movers: AOR = 0.72; CI = 0.63–0.83), translating into a predicted probability of 38.4% for high-movers, 45.1% for low-movers, and 53.2% for non-movers.

Results were somewhat uneven with regard to exposure to violence and harassment as a victim (Table 3). The odds of being hit or pushed were significantly higher for both high-movers (AOR = 1.34; CI = 1.17–1.54) and low-movers (AOR = 1.26; CI = 1.17–1.37). However, the odds of having rumors or lies spread about them were significantly higher only for high-movers (AOR = 1.40; CI = 1.22–1.60), while the odds of being teased about their body was significantly higher only for low-movers (AOR = 1.14; CI = 1.06–1.24). Among the three measures of victimization, for both movers and non-movers, predicted probabilities were greatest for having rumors or lies spread (high-movers: 46.3%; low-movers: 40.7%; non-movers: 38.2%).

Both low and high moving were associated with increased odds of perpetuating violence or harassment (Table 3). Compared to non-movers, high-movers had greater odds of hitting or pushing a classmate (AOR = 1.54; CI = 1.24–1.92), translating into a 35.3% predicted probability, compared to 30.0% for low-movers and 26.1% for non-movers. High-movers also had greater odds of spreading rumors or lies about a classmate (AOR = 1.59; CI = 1.28–1.97), with a predicted probability of 28.4%, compared to 25.5% for low-movers and 20.0% for non-movers.

## Discussion

There was a high level of residential mobility among this study’s sample. Over one in three respondents reported moving in the past year. These findings exceed recent national estimates, which indicate that 11% of youth move in a given year (U.S. Census Bureau, 2018). The high level of residential mobility may partially reflect contextual elements of the study’s urban setting, such as the concentration of renters in urban areas (Joint Center for Housing Studies of Harvard University, 2013), and the dwindling local supply of affordable housing to rent or own (California Housing Partnership Corporation, 2018). The high prevalence of moving observed in this study – especially frequent moving – suggests that educators, especially in urban public school districts, should recognize residential mobility as a potentially common issue among their students, particularly given the associations observed with poor school experiences.

In general, modest dose–response relationships in expected directions were observed between level of residential mobility and a range of negative school experiences. Movers, especially high-movers, had poorer school experiences than non-movers. Previous work has documented the relationship between moving and poor distal academic outcomes, like absenteeism and dropout (Blum, 2005; Voight et al., 2012; Metzger et al., 2015; National Collaborative on Education and Health, 2015). This study augments that work by documenting how more proximal negative school experiences may fit on the pathway linking residential mobility to school failure, in line with this study’s theoretical framework (Figure 1). Specifically, the present study provides additional nuance regarding how residential mobility at early ages may relate to negative academic trajectories. Namely, in adjusted models, inverse relationships between residential mobility and school connectedness or caring teachers/staff were only observed for high-movers, contrary to the expectation that this relationship would also be observed among low-movers. Similarly, not all measures of exposure to violence or harassment as a victim exhibited a clear dose–response relationship with residential mobility, whereas this relationship was present for measures of perpetration of school violence or harassment, as well as perceived academic ability. One interpretation is that the act of moving homes may cause stress for young children, or compound stress stemming from other factors. Among low-movers, this stress could manifest as aggressive behavior and a poor perception of one’s academic ability, but may not necessarily erode relationships or provoke bullying from other students. Meanwhile, among high-movers, this stress could additionally manifest as low levels of school connectedness and perceived lack of caring teachers/staff, possibly because frequent moving could also mean moving schools, and/or could reflect greater stress occurring in the student’s home life.

These findings highlight several opportunities for educators to prevent or intervene on negative academic trajectories by paying closer attention to residential mobility. Recognizing the importance of moving could be beneficial for one simple reason: schools may be alerted that a student has moved, but never be informed of underlying issues. Evidence points to the close relationship between socioeconomic vulnerability (e.g., poverty, structural racism), associated neighborhood and family factors (e.g., caretaker instability, exposure to in-home or neighborhood violence, and poor personal or family health), and both residential mobility and academic success (Figure 1; Acevedo-Garcia et al., 2008; Jelleyman and Spencer, 2008; Herbers et al., 2012; Perkins, 2017). Tracking residential mobility may be a way to help schools identify students with an elevated risk of experiencing these issues. If caretakers update their address with the school office when a move occurs, schools would know about a student’s residential mobility. Many schools already have protocol in place to proactively support students who have changed schools; the present findings indicate that implementing similar mechanisms to identify and engage students who move homes may be a valuable strategy to prevent academic problems. Given inevitable resource constraints, schools may want to prioritize high-movers, or alternately, flag all movers, and monitor for early signs of trouble, including increased perpetration of violence. Finally, even among high-movers, close to four out of five respondents reported feeling that there was an adult at school who cared about them all the time. Interventions designed to support residentially mobile youth could leverage this critical protective factor (National Center on Safe Supportive Learning Environments, 2019) to stabilize school-based relationships and respond to problematic behavior early on, potentially preventing subsequent problems with absenteeism and poor academic performance that can compound over a student’s academic life.

### Limitations and Next Steps

Despite providing preliminary information to understand the relationship between residential mobility and poor school experiences, this study has several limitations. First, our theoretical framework highlights the complex relationship between socioeconomic and demographic characteristics, neighborhood and family factors, residential mobility, and school experiences. While these factors are heavily intertwined (Eisenberg et al., 2003; Carbone-Lopez et al., 2010; Morrissey et al., 2013; Voight et al., 2015), only some of these variables could be controlled for in the current analysis (because they were present in the dataset). Notably, although this study’s descriptive analysis of district characteristics suggests that districts in the analysis had a generally comparable socioeconomic and demographic profile to that of other districts in Los Angeles County, it was not possible to control for these factors at the student-level. Furthermore, the study could not differentiate between students who moved homes and those who moved both homes and schools (recent estimates suggest an approximate 40% overlap between these groups) (Voight et al., 2012, 2017). Second, the sequencing of the home move relative to the school experiences analyzed here is not understood. For example, it was not known whether a mover who reported having been hit or pushed experienced this victimization before or after their move. Relatedly, the CHKS is typically administered in the fall or spring; questions that ask students to report on “past year” incidents may have been interpreted by students to refer either to the last 12 months, or the previous academic year. Third, because the data were obtained from a cross-sectional survey, determining the direction of relationships between variables under study was not possible (Gasper et al., 2010). Fourth, not all districts in Los Angeles County completed the elementary CHKS in the study year and not all districts in the sample collected data from 100% of students; little information is available to indicate if schools followed sampling guidance, although WestEd’s standards for minimally acceptable response rates were incorporated into inclusion criteria for this study’s analysis sample. While the sample size was large, the results may not be generalizable to other communities and contexts; caution is especially warranted when interpreting findings outside of the United States. Fifth, all variables were measured via student self-report, and therefore may be susceptible to recall and social desirability bias.

Additional research is needed to better characterize the relationship between residential mobility and its influence on negative academic trajectories, including potential intermediate outcomes like school disconnectedness and absenteeism. First, there is a need for studies of elementary school students that include relevant student-level characteristics not available in the present analysis, such as (but not limited to) race/ethnicity, a measure of household income, and school mobility (Garboden et al., 2017). Second, more longitudinal studies on this topic are needed to determine the sequencing of the residential move(s) relative to the negative school experiences, and to identify potential cumulative effects and analyze causal associations. Third, mixed methods studies or qualitative methods are needed to confirm or contrast patterns observed in the present analysis; interviews with students or teachers/staff could provide rich context to quantitative findings. A more robust understanding of the relationship between residential mobility and school experiences can help schools design and evaluate strategies to identify and support mobile students, potentially providing a valuable lever to prevent or interrupt the pathway toward school disconnectedness, absenteeism, and academic failure.

## Data Availability Statement

The individual student-level dataset analyzed in this study was purchased from WestEd. Per the memorandum of understanding in connection with this dataset, the authors are not permitted to share this dataset. Requests to access the dataset should be directed to WestEd.

## Ethics Statement

The study was deemed exempt from review by the Institutional Review Board of the Los Angeles County Department of Public Health. Written informed consent to participate in data collection for this study was provided by the participants’ legal guardian/next of kin.

## AUTHOR CONTRIBUTIONS

GG and AD contributed to the design of the study and wrote the sections of the manuscript. With contributions from AD, GG cleaned the dataset, performed the statistical analysis, and interpreted the results. GG, AD, and TK contributed to the manuscript revision, and read and approved the submitted version of the manuscript.

## Conflict of Interest

The authors declare that the research was conducted in the absence of any commercial or financial relationships that could be construed as a potential conflict of interest.

## References

[B1] Acevedo-Garcia D. Osypuk T. L. McArdle N. Williams D. R. (2008). Toward a policy-relevant analysis of geographic and racial/ethnic disparities in child health. *Health Aff.* 27 321–333. 10.1377/hlthaff.27.2.321 18332486

[B2] Adams J. M. (2013). *New Focus on School Climate in mAssive Student Survey.* Available at: https://edsource.org/2013/revised-student-survey-reflects-focus-on-school-climate/39243 (accessed June 18, 2019).

[B3] Amaral G. Geierstanger S. Soleimanpour S. Brindis C. (2011). Mental health characteristics and health-seeking behaviors of adolescent school-based health center users and nonusers. *J. Sch. Health* 81 138–145. 10.1111/j.1746-1561.2010.00572.x 21332478

[B4] Anderson S. Leventhal T. Newman S. Dupéré V. (2014). Residential mobility among children: a framework for child and family policy. *Cityscape* 16 5–36.

[B5] Armstrong K. E. Bush H. M. Jones J. (2010). Television and video game viewing and its association with substance use by Kentucky elementary school students, 2006. *Public Health Rep.* 125 433–440. 10.1177/003335491012500312 20433038 PMC2848268

[B6] Ashley K. M. Ennis L. S. Owusu-Ansah A. (2012). An exploration of middle school students’ perceptions of personal adolescent wellness and their connectedness to school. *Int. J. Soc. Sci. Educ.* 2 74–89.

[B7] Astone N. M. McLanahan S. S. (1994). Family structure, residential mobility, and school dropout: a research note. *Demography* 31 575–584. 7890092

[B8] Austin G. (2013). *The California Healthy Kids Survey: The Case for Continuation.* Available at: https://data.calschls.org/resources/PreventionTactics.pdf (accessed June 18, 2019).

[B9] Austin G. Bates S. Duerr M. (2013). *Guidebook to the California Healthy Kids Survey: Part II Survey Content Core Module.* Available at: https://data.calschls.org/resources/chks_guidebook_2_coremodules.pdf (accessed June 18, 2019).

[B10] Beck B. Buttaro A. Jr. Lennon M. C. (2016). Home moves and child wellbeing in the first five years of life in the United States. *Longit. Life Course Stud.* 7 240–264.

[B11] Blum R. W. (2005). A case for school connectedness. *Adolesc. Learner* 62 16–20.

[B12] Bostean G. Trinidad D. R. McCarthy W. J. (2015). E-cigarette use among never-smoking California students. *Am. J. Public Health* 105 2423–2425. 10.2105/AJPH.2015.302899 26469671 PMC4638243

[B13] California Department of Education (2019). *Safe and Supportive Schools.* Available at: https://www.cde.ca.gov/ls/ss/se/safesupportive.asp (accessed June 18, 2019).

[B14] California Healthy Kids Survey (2019). *California School Climate, Health, and Learning Surveys.* Available at: http://chks.wested.org (accessed June 18, 2019).

[B15] California Housing Partnership Corporation (2018). *Los Angeles County Annual Affordable Housing Outcomes Report.* Available at: https://1p08d91kd0c03rlxhmhtydpr-wpengine.netdna-ssl.com/wp-content/uploads/2018/06/Full-LA-County-Outcomes-Report-with-Appendices.pdf (accessed June 18, 2019).

[B16] Carbone-Lopez K. Finn-Aage E. Brick B. T. (2010). Correlates and consequences of peer victimization: gender differences in direct and indirect forms of bullying. *Youth Violence Juv Justice* 8 332–350. 10.1177/1541204010362954

[B17] Cederbaum J. A. Gilreath T. D. Benbenishty R. Astor R. A. Pineda D. DePedro K. T. (2014). Well-being and suicidal ideation of secondary school students from military families. *J. Adolesc. Health* 54 672–677. 10.1016/j.jadohealth.2013.09.006 24257031

[B18] Centers for Disease Control and Prevention (2009). *School Connectedness: Strategies for Increasing Protective Factors Among Youth.* Available at: https://www.cdc.gov/healthyyouth/protective/pdf/connectedness.pdf (accessed June 18, 2019).

[B19] Centers for Disease Control and Prevention (2018). *School Connectedness.* Available at: https://www.cdc.gov/healthyyouth/protective/school_connectedness.htm (accessed June 18, 2019).

[B20] Child and Family Research Partnership (2017). *5 Things you Should Know About Military-Connected Youth.* Available at: https://childandfamilyresearch.utexas.edu/sites/default/files/201707_5-things_MilitaryYouth.pdf (accessed June 18, 2019).

[B21] Coulton C. Theodos B. Turner M. A. (2012). Residential mobility and neighborhood change: real neighborhoods under the microscope. *Cityscape* 14 55–89.

[B22] Davis A. M. Kreutzer R. Lipsett M. King G. Shaikh N. (2006). Asthma prevalence in Hispanic and Asian American ethnic subgroups: results from the California Healthy Kids Survey. *Pediatrics* 118 e363–e370. 10.1542/peds.2005-2687 16882779

[B23] Davis A. M. Lipsett M. Milet M. Etherton M. Kreutzer R. (2007). An association between asthma and BMI in adolescents: results from the California Healthy Kids Survey. *J. Asthma* 44 873–879. 10.1080/02770900701752656 18097866

[B24] Department of Education (2019). *Chronic Absenteeism in the Nation’s schools: A Hidden Educational Crisis.* Available at: https://www2.ed.gov/datastory/chronicabsenteeism.html (accessed June 18, 2019).

[B25] Dong M. Ada R. F. Felitti V. J. Williamson D. F. Dube S. R. Brown D. W. (2005). Childhood residential mobility and multiple health risks during adolescence and adulthood: the hidden role of adverse childhood experiences. *Arch Pediatr Adolesc Med.* 159 1104–1110. 16330731 10.1001/archpedi.159.12.1104

[B26] Eisenberg M. E. Neumark-Sztainer D. Perry C. L. (2003). Peer harassment, school connectedness, and academic achievement. *J. Sch. Health* 73 311–316. 10.1111/j.1746-1561.2003.tb06588.x 14593947

[B27] Ersing R. L. Sutphen R. D. Loeffler D. N. (2009). Exploring the impact and implications of residential mobility: from the neighborhood to the school. *Advan. Soc. Work* 10 1–18. 10.18060/77

[B28] Estrada J. N. Gilreath T. D. Astor R. A. Benbenishty R. (2013). Gang membership of California middle school students: behaviors and attitudes as mediators of school violence. *Health Educ Res.* 24 626–639. 10.1093/her/cyt037 23525778

[B29] Felix E. D. Furlong M. J. Austin G. (2009). A cluster analytic investigation of school violence victimization among diverse students. *J. Interpers. Violence* 24 1673–1695. 10.1177/0886260509331507 19252063

[B30] Freudenberg N. Ruglis J. (2007). Reframing school dropout as a public health issue. *Prev. Chronic Dis.* 4 A107. 17875251 PMC2099272

[B31] Garboden P. M. E. Leventhal T. Newman S. (2017). Estimating the effects of residential mobility: a methodological note. *J. Soc. Serv. Res.* 43 246–261. 10.1080/01488376.2017.1282392

[B32] Gasper J. DeLuca S. Estacion A. (2010). Coming and going: explaining the effects of residential and school mobility on adolescent delinquency. *Soc. Sci. Res.* 39 459–476. 10.1016/j.ssresearch.2009.08.009

[B33] Gastic B. (2008). School truancy and the disciplinary problems of bullying victims. *Educ. Rev.* 60 391–404. 10.1080/00131910802393423

[B34] Gee K. Krausen K. (2015). *Safety Linked to Reduced Truancy in High-Poverty Schools.* Available at: https://poverty.ucdavis.edu/policy-brief/safety-linked-reduced-truancy-high-poverty-schools (accessed on June 18, 2019).

[B35] Gilreath T. D. Astor R. A. Cederbaum J. A. Atuel H. Benbenishty R. (2014a). Prevalence and correlates of victimization and weapon carrying among military- and nonmilitary-connected youth in Southern California. *Prev. Med.* 60 21–26. 10.1016/j.ypmed.2013.12.002 24333605

[B36] Gilreath T. D. Astor R. A. Estrada J. N. Jr. Benbenishty R. Unger J. B. (2014b). School victimization and substance use among adolescents in California. *Prev. Sci.* 15 897–906. 10.1007/s11121-013-0449-8 24482139 PMC5087280

[B37] Gilreath T. D. Cederbaum J. A. Astor R. A. Benbenishty R. Pineda D. Atuel H. (2013). Substance use among military-connected youth: the California Healthy Kids Survey. *Am. J. Prev. Med.* 44 150–153. 10.1016/j.amepre.2012.09.059 23332331

[B38] Herbers J. E. Cutuli J. J. Supkoff L. M. Heistad D. Chan C.-K. Hinz E. (2012). Early reading skills and academic achievement trajectories of students facing poverty, homelessness, and high residential mobility. *Educ. Res.* 41 366–374. 10.3102/0013189x12445320

[B39] Jelleyman T. Spencer N. (2008). Residential mobility in childhood and health outcomes: a systematic review. *J. Epidemiol. Community Health* 62 584–592. 10.1136/jech.2007.060103 18559440

[B40] Joint Center for Housing Studies of Harvard University (2013). *The State of the Nation’s Housing, 2013.* Available at: https://www.jchs.harvard.edu/sites/default/files/son2013_0.pdf (accessed on June 18, 2019).

[B41] Juvonen J. Wang Y. Espinoza G. (2011). Bullying experiences and compromised academic performance across middle school grades. *J Early Adolesc.* 31 152–173. 10.1177/0272431610379415

[B42] Kang S. (2019). Why low-income households become unstably housed: evidence from the panel study of income dynamics. *Hous Policy Debate* 29 559–587. 10.1080/10511482.2018.1544161

[B43] Kearney C. A. (2008). School absenteeism and school refusal behavior in youth: a contemporary review. *Clin. Psychol. Rev.* 28 451–471. 10.1016/j.cpr.2007.07.012 17720288

[B44] Ladd G. W. Ettekal I. Kochenderfer-Ladd B. (2017). Peer victimization trajectories from kindergarten through high school: differential pathways for children’s school engagement and achievement? *J. Educ. Psychol.* 109 826–841. 10.1037/edu0000177

[B45] Lawrence E. Root E. D. Mollborn S. (2015). Residential mobility in early childhood: household and neighborhood characteristics of movers and non-movers. *Demogr Res.* 33 939–950. 10.4054/demres.2015.33.32 26819568 PMC4724801

[B46] Lenzi M. Sharkey J. Vieno A. Mayworm A. Dougherty D. Nylund-Gibson K. (2015). Adolescent gang involvement: the role of individual, family, peer, and school factors in a multilevel perspective. *Aggress. Behav.* 41 386–397. 10.1002/ab.21562 25288165

[B47] Lewis C. Deardorff J. Lahiff M. Soleimanpour S. Sakashita K. Brindis C. D. (2015). High school students’ experiences of bullying and victimization and the association with school health center use. *J. Sch. Health* 85 318–326. 10.1111/josh.12256 25846311

[B48] Low J. A. Hallett R. E. Mo E. (2017). Doubled-up homeless: comparing educational outcomes with low-income students. *Educ. Urban Soc..* 49 795–813. 10.1177/0013124516659525

[B49] Marsh H. W. Martin A. J. (2011). Academic self-concept and academic achievement: relations and causal ordering. *Br. J. Educ. Psychol.* 81 59–77. 10.1348/000709910X503501 21391964

[B50] Metzger M. W. Fowler P. J. Anderson C. L. Lindsay C. A. (2015). Residential mobility during adolescence: do even “upward” moves predict dropout risk? *Soc. Sci. Res.* 53 218–230. 10.1016/j.ssresearch.2015.05.004 26188449 PMC4508758

[B51] Mmari K. N. Bradshaw C. P. Sudhinaraset M. Blum R. (2010). Exploring the role of social connectedness among military youth: perceptions from youth, parents, and school personnel. *Child Youth Care Forum.* 39 351–366. 10.1007/s10566-010-9109-3

[B52] Mollborn S. Lawrence E. Root E. D. (2018). Residential mobility across early childhood and children’s kindergarten readiness. *Demography* 55 485–510. 10.1007/s13524-018-0652-0 29492798 PMC5898794

[B53] Morrissey T. W. Hutchison L. Winsler A. (2013). Family income, school attendance, and academic achievement in elementary school. *Dev. Psychol.* 50 741–753. 10.1037/a0033848 23914750

[B54] Murphey D. Bandy T. Moore K. A. (2012). *Frequent Residential Mobility and Young Children’s Well-Being.* Available at: https://www.childtrends.org/wp-content/uploads/2012/01/Child_Trends-2012_02_14_RB_Mobility.pdf (accessed on June 18, 2019).

[B55] National Center on Safe Supportive Learning Environments (2019). *Protective Factors.* Available at: https://safesupportivelearning.ed.gov/training-technical-assistance/education-level/early-learning/protective-factors (accessed on June 18, 2019).

[B56] National Collaborative on Education and Health (2015). *Brief on Chronic Absenteeism and School Health.* Available at: https://www.attendanceworks.org/wp-content/uploads/2017/09/Chronic-Absenteeism-and-School-Health-Brief-1.pdf (accessed on June 18, 2019).

[B57] National Education Association (2019). *Facts About Child Nutrition* Available at: http://www.nea.org/home/39282.htm (accessed on June 18, 2019).

[B58] O’Malley M. Voight A. Renshaw T. L. Eklund K. (2015). School climate, family structure, and academic achievement: a study of moderation effects. *Sch. Psychol. Q.* 30 142–157. 10.1037/spq0000076 25111464

[B59] Perez-Brumer A. Day J. K. Russell S. T. Hatzenbuehler M. L. (2017). Prevalence and correlates of suicidal ideation among transgender youth in California: findings from a representative, population-based sample of high school students. *J. Am. Acad. Child Adolesc. Psychiatry* 56 739–746. 10.1016/j.jaac.2017.06.010 28838578 PMC5695881

[B60] Perkins K. L. (2017). Reconsidering residential mobility: differential effects on child wellbeing by race and ethnicity. *Soc. Sci. Res.* 63 124–137. 10.1016/j.ssresearch.2016.09.024 28202137

[B61] Quin D. (2017). Longitudinal and contextual associations between teacher-student relationships and student engagement: a systematic review. *Rev Educ Res.* 87 345–387. 10.3102/0034654316669434

[B62] Russell S. T. Sinclair K. O. Poteat P. Koenig B. W. (2012). Adolescent health and harassment based on discriminatory bias. *Am. J. Public Health* 102 493–495. 10.2105/AJPH.2011.300430 22390513 PMC3487669

[B63] Scanlon E. Devine K. (2001). Residential mobility and youth well-being: research, policy, and practice issues. *J. Soc. Soc. Welfare* 28 119–139.

[B64] Sheryl A. H. Stephanie M. P. Herrenkohl T. I. Toumbourou J. W. Catalano R. F. (2014). Student and school factors associated with school suspension: a multilevel analysis of students in Victoria, Australia and Washington State, United States. *Child Youth Serv. Rev.* 36 187–194. 10.1016/j.childyouth.2013.11.022 24860205 PMC4028069

[B65] Skobba K. Goetz E. G. (2013). Mobility decisions of very low-income households. *Cityscape* 15 155–171.

[B66] Sparshott J. (2015). *Rising Rents Outpace Wages in Wide Swaths of the U.S.* Available at: https://www.wsj.com/articles/rising-rents-outpace-wages-in-wide-swaths-of-the-u-s-1438117026 (accessed on June 18, 2019).

[B67] Spriggs A. L. Iannotti R. J. Nansel T. R. Haynie D. L. (2007). Adolescent bullying involvement and perceived family, peer and school relations: commonalities and differences across race/ethnicity. *J. Adolesc. Health* 41 283–293. 10.1016/j.jadohealth.2007.04.009 17707299 PMC1989108

[B68] Steiner R. J. Rasberry C. N. (2015). Brief report: associations between in-person and electronic bullying victimization and missing school because of safety concerns among U.S. high school students. *J. Adolesc.* 43 1–4. 10.1016/j.adolescence.2015.05.005 26043166 PMC9125422

[B69] Stone S. Whitaker K. Anyon Y. Shields J. P. (2013). The relationship between use of school-based health centers and student-reported school assets. *J. Adolesc. Health* 53 526–532. 10.1016/j.jadohealth.2013.05.011 23849547

[B70] Sullivan K. Capp G. Gilreath T. D. Benbenishty R. Roziner I. Astor R. A. (2015). Substance abuse and other adverse outcomes for military-connected youth in California: results from a large-scale normative population survey. *JAMA Pediatr.* 169 922–928. 10.1001/jamapediatrics.2015.1413 26280338

[B71] Turner M. A. Ross S. L. (2005). “How racial discrimination affects the search for housing,” in *The Geography of. (Opportunity): Race and Housing Choice in Metropolitan America*, ed. de Souza Briggs X. (Washington, DC: Institution Press), 81–100.

[B72] Tyler J. H. Lofstrom M. (2009). Finishing high school: alternative pathways and dropout recovery. *Future Child.* 19 77–103. 10.1353/foc.0.0019 21141706

[B73] U.S. Census Bureau (2018). *Table 1. General Mobility, by Race and Hispanic Origin and Region, and by Sex, Age, Relationship to Householder, Educational Attainment, Marital Status, Nativity, Tenure, and Poverty Status: 2017 to 2018. Current Population Survey, 2018 Annual Social and Economic Supplement.* Available at: https://www.census.gov/data/tables/2018/demo/geographic-mobility/-2018.html (accessed on June 18, 2019).

[B74] U.S. Department of Health and Human Services (2019). *Effects of Bullying.* Available at: https://www.stopbullying.gov/at-risk/effects/index.html (accessed on June 18, 2019).

[B75] U.S. Department of Housing and Urban Development (2014). *How Housing Mobility Affects Education Outcomes for Low-Income Children.* Available at: https://www.huduser.gov/portal/periodicals/em/fall14/highlight2.html (accessed August 19, 2019).

[B76] Voight A. Giraldo-Garcia R. Shinn M. (2017). The effects of residential mobility on the education outcomes of urban middle school students and the moderating potential of civic engagement. *Urban Educ.* 1–22.

[B77] Voight A. Hanson T. O’Malley M. Adekanye L. (2015). The racial school climate gap: within-school disparities in students’ experiences of safety, support, and connectedness. *Am. J. Community Psychol.* 56 252–267. 10.1007/s10464-015-9751-x 26377419

[B78] Voight A. Shinn M. Nation M. (2012). The longitudinal effects of residential mobility on the academic achievement of urban elementary and middle school students. *Educ. Res.* 41 385–392. 10.3102/0013189x12442239

[B79] WestEd (2014). *Search LEA Reports.* Available at: https://calschls.org/reports-data/search-lea-reports/ (accessed on June 18, 2019).

[B80] WestEd (2019). *California Healthy Kids Survey* Available at: https://www.wested.org/project/california-healthy-kids-survey-chks/ (accessed on June 18, 2019).

[B81] Wong M. M. Klingle R. S. Price R. K. (2004). Alcohol, tobacco, and other drug use among Asian American and Pacific Islander Adolescents in California and Hawaii. *Addict. Behav.* 29 127–141. 10.1016/s0306-4603(03)00079-0 14667425

[B82] Woolley M. E. Grogan-Kaylor A. (2006). Protective family factors in the context of neighborhood: PROMOTING positive school outcomes. *Fam Relat.* 55 93–104. 10.1111/j.1741-3729.2006.00359.x

[B83] Ziol-Guest K. M. McKenna C. C. (2014). Early childhood housing instability and school readiness. *Child Dev.* 85 103–113. 10.1111/cdev.12105 23534607

